# The Financial Burden of Cancer on Families in the United States

**DOI:** 10.3390/ijerph18073790

**Published:** 2021-04-05

**Authors:** Patrick Richard, Nilam Patel, Yuan-Chiao Lu, Regine Walker, Mustafa Younis

**Affiliations:** 1 Department of Preventive Medicine and Biostatistics, Uniformed Services University of the Health Sciences, Bethesda, MD 20814, USA; 2 The Henry M. Jackson Foundation for the Advancement of Military Medicine, Bethesda, MD 20814, USA; nilam.patel.ctr@usuhs.edu (N.P.); vtyclu@gmail.com (Y.-C.L.); regine.walker.ctr@usuhs.edu (R.W.); 3 Department of Health Policy and Management, School of Public Health, Jackson State University, Jackson, MS 39217, USA; younis99@gmail.com

**Keywords:** cancer, medical debt, out of pocket costs, panel study of income dynamics

## Abstract

This study examined the relationship between a diagnosis of cancer and the likelihood of having any out-of-pocket costs (OOPC) and medical debt, and the amounts of OOPC and medical debt, at the household level. We used the 2013 Panel Study of Income Dynamics, a continuous, representative panel survey that collects demographic, economic, and social data in the United States. The analytic sample included head of households and their spouse (if married), 18–64 years old. Two-part models were used. The first part consisted of logistic regression models and the second part consisted of generalized linear models with logarithmic link and a gamma distribution. Logistic regression results showed odds of 2.13 (CI: 1.27, 3.57, *p* < 0.01) for any OOPC and odds of 1.55 (CI: 0.93, 2.58, *p* < 0.1) for any medical debt for households in which either the head or spouse (if married) reported a diagnosis of cancer compared to those that did not report a diagnosis of cancer. Likewise, results from the second part of the model for households with a positive amount of OOPC showed an exponentiated coefficient of 1.73 (CI: 1.33, 2.25, *p* < 0.01) for households in which either the head or spouse (if married) reported a diagnosis of cancer compared to households without a diagnosis of cancer. This study shows that a diagnosis of cancer places a financial burden on families, particularly with all types of debt, in the United States even after controlling for differences between households with a diagnosis of cancer and those without a diagnosis of cancer.

## 1. Introduction

The financial burden of cancer on individuals and their families is anticipated to rise substantially in the United States because of the increase in cost sharing associated with new and expensive cancer treatment technology [1,2,3,4,5,6]. High out-of-pocket costs (OOPC) have contributed to medical debt for patients with cancer, even for those who have health insurance [7,8]. Several studies have found that the financial impact of cancer on patients and families may include poorer quality of life, changes in receiving medical care, or even declaring bankruptcy [9,10,11,12].

A diagnosis of cancer may place a financial burden on patients and families through large and repeated costs of treatment [13,14,15,16,17,18,19,20]. The literature in this area has consistently found that a catastrophic health event such as a diagnosis of cancer is associated with large OOPC for both elderly and non-elderly individuals [13,15,16,17,18,19,20,21,22]. However, the literature on the impact of chronic conditions, such as a diagnosis of cancer, at the household level is limited [23]. Further, while socioeconomic status has been used to evaluate the financial burden of health conditions (see [24,25,26]), there is a limited understanding of the impact of cancer on medical debt. It is important to consider medical debt related to a diagnosis of cancer, which may for example also have a consequence of filing for bankruptcy [27].

A diagnosis of cancer may also impose a financial burden on households through a loss of employment by either the head of household or a spouse diagnosed with cancer. Households in which family members have been diagnosed with cancer may experience a “productivity” shock [28]. That is, a household member diagnosed with cancer may not be able to participate in the labor markets because of the time needed for treatment and other treatment-related activities, which may result in loss of health insurance coverage [29]. Such a loss of health insurance would increase OOPC and medical debt [7]. Similar to this study, household level analyses also control for relevant household characteristics such as the number of dependent children in the household and whether a spouse has insurance coverage, which have been found to be associated with the outcomes under study [30]. Individual level analyses only control for the patient’s socio-demographic and clinical characteristics (e.g., [20]).

The previously mentioned studies provide information on the impact of a diagnosis of cancer on OOPC and employment and the role of health insurance in linking these variables. Close to our study, Davidoff et al. [20] investigated the out of pocket burden faced by Medicare beneficiaries with cancer and found that Medicare beneficiaries with cancer spent an additional $976 annually compared to those without cancer. In contrast to Davidoff et al. [20], the current study focuses on the non-elderly population aged 18–64 years and includes medical debt as another measure of financial burden. The current study also uses the household as the unit of analysis, which is important in informing policies that target the financial well-being of households as opposed to individuals.

## 2. Materials and Methods

### 2.1. Data Source and Participants

Panel Study of Income Dynamics (PSID) [31] data from 2013 were used. The PSID is a continuous, representative panel survey that collects demographic, economic, and social data from individuals and families in the United States. The unique advantage of using the PSID to complete the current analysis, compared to other data sets such as the Medical Expenditure Panel Survey (MEPS), is that financial resources such as medical debt at the household level is captured. The PSID also has information on the type of debt (i.e., credit card, student loan, medical, legal, mortgage, car loan, and loans from relatives). Additionally, the PSID has some chronic conditions, such as bipolar disorder, lung disease, and schizophrenia, relevant to the current study, that the MEPS for example does not have available in the household component file.

The 2013 wave of the PSID contains data on 9063 households. Households were used as the unit of analysis (see [30] for further details). Additionally, although we only observed the cancer status for the head of household and spouse (if married), other family members such as children could be impacting financial burden. The head of household may acquire medical debt for family members such as children because of the utility he or she would receive [32,33]. To address this issue, the number of children was controlled for. Further, households with family members who are part of a health insurance eligibility unit were included and those that had non-family members (e.g., friends, nephews) living in the home were not included.

Only households whose head of household and spouse (if married) were 18 to 64 years old were included in the analysis (based on this criterion, 1112 households were excluded). Households that did not form a health insurance eligibility unit were excluded (1150 households). “Dual eligibles” or beneficiaries of both Medicare and Medicaid were not included (52 households) because of special health needs that may impact financial outcomes. Further, only households with debt (e.g., credit card, medical, etc.) were included (based on this criterion, 1960 households were excluded). By only including households with debt, medical debt as a proportion of total debt can be observed. In order to ensure that the diagnosis of cancer occurred before the financial outcomes, we excluded households that reported a diagnosis of cancer in 2011, 2012, or 2013 (72 households). These measurements were used in determining the majority of the households that were removed, with the other removed households having missing data (310 households). For the dependent variables of any OOPC, outcome 1, and any medical debt, outcome 2, there was an analytical sample of 4407 households. Of note, those with $0 of debt were included as having any OOPC and any medical debt [23,30,34]. Further, consistent with the literature, households with positive amounts of OOPC and medical debt were used to investigate the relationship between a diagnosis of cancer and the amount of OOPC, outcome 3, and the amount of medical debt, outcome 4. This resulted in a sample size of 3183 households with positive amounts of OOPC and 639 households with positive amounts of medical debt.

### 2.2. Measurement

#### 2.2.1. Dependent Variables

To provide an assessment of the financial burden that a diagnosis of cancer might impose on families, four dependent variables were used: (1) any OOPC; (2) any medical debt; (3) amount of OOPC (OOPC > $0); (4) amount of medical debt (medical debt > $0). “Any OOPC” and “any medical debt” was coded as 1 to indicate the presence of any OOPC or any medical debt and 0 if not present. The variables amount of OOPC and medical debt were treated as continuous. Participant’s responses to two questions were used to determine OOPC: “About how much did (you (and your family)/they) pay out-of-pocket for nursing home and hospital bills in 2012?” and “About how much did you (and your family) pay out-of-pocket for doctor, outpatient surgery, and dental bills in 2011 and 2012 combined?” Similar to the National Health Interview Survey [7,35], the PSID measures medical debt by using the following question: “If you added up all medical bills [respondents/family members living there], about how much would they amount to right now? INCLUDE unpaid balance(s), or medical bills that are outstanding.” In other words, only unpaid balances from receiving medical treatment were included as “medical debt”. The questions above that were used to determine OOPC asked about costs for either 2012 or 2011 and 2012 and were thus adjusted to 2013 U.S. dollars. There was no need to adjust medical debt to 2013 dollars because the question asked participants for the amount at the current time.

#### 2.2.2. Independent Variables

A self-reported diagnosis of cancer of the head of household or spouse was the key independent variable. The self-reported diagnosis was based on the participant ever being told by a doctor or health professional that he or she has cancer or a malignant tumor. All stages of cancer and all types of cancer (i.e., bladder cancer, breast cancer, colon cancer, lung cancer, prostate cancer, cervical cancer, lymphoma or leukemia, ovarian cancer, skin cancer—melanoma, skin cancer—non melanoma (e.g., basal cell), skin cancer—DK type, thyroid cancer, uterine cancer, other) were included. The models controlled for a vector of variables based on the literature [36,37,38,39,40]. These were the head of household’s sex, categorical age, race, marital status, and years of education. Years of education squared was also included in order to capture the non-linear relationship between years of education and financial outcomes. Household variables included the number of children in the household, the number of chronic health conditions for the head of household and spouse if married, health insurance status, geographic region, and geographic variation. Chronic health conditions were determined by the question, “Has a doctor or other health professional EVER told (you/HEAD) that (you/he/she) had...CONDITION?”. The chronic health conditions were arthritis, asthma, bipolar disorder, diabetes, heart attack, heart disease, hypertension, lung disease, schizophrenia and stroke. Geographic region was included to account for geographic variances in coverage of health insurance and OOPC and medical debt. The number of chronic health conditions was log transformed because of the non-normal distribution of the variable. Finally, we used family weights to account for non-responses and oversampling in the data [31].

### 2.3. Statistical Analysis

Logistic regression models were used to compute the odds ratios of having any OOPC and any medical debt between households that (either the head of household or spouse if married) reported a diagnosis of cancer and those who did not report a diagnosis of cancer. Odds ratios are presented for easier interpretation of the results. For households with positive amounts of OOPC and medical debt, we estimated generalized linear models (GLMs) with logarithmic link and a gamma distribution to investigate the relationship between households in which members reported a diagnosis of cancer and those that did not report a diagnosis of cancer and the amounts of OOPC and medical debt. Exponentiated coefficients are presented for easier interpretation of the results. All tests of significance were two-sided.

## 3. Results

The weighted descriptive results are presented in Table 1. Summary statistics are presented on a total sample of 4407 households with any OOPC or any medical debt (first part of the model). Over 5% (*n* = 242) of the households in the sample had either the head of household or spouse with a diagnosis of cancer. About 78% (*n* = 3437) of households had any OOPC. Further, significant differences in the likelihood of any OOPC for households with a diagnosis of cancer compared to those without any diagnosis of cancer were found (91% vs. 78%, *p* < 0.001). About 12% (*n* = 529) of households had any medical debt. Unadjusted results found no significant differences in the likelihood of having any medical debt between households in which members reported a diagnosis of cancer compared to those that did not report a diagnosis of cancer (14% vs. 12%, *p* = 0.4289).

In Table 2, weighted summary statistics are presented for households with positive amounts of OOPC (*n* = 3183) and medical debt (*n* = 639) (second part of the model). Results showed that the average amount of OOPC per household for households with positive amounts of OOPC was $1061 in 2013 dollars. This amount of OOPC is similar to the annual amount of OOPC found in studies that use the MEPS [1,2,3]. Statistically significant differences were found in the average annual amount of OOPC per household between households with a diagnosis of cancer compared to those without any diagnosis of cancer ($1766 vs $1011, *p* < 0.001). For households with positive amounts of medical debt, results in Table 2 showed that the average amount of medical debt per household was $9023 in 2013 dollars and no differences were found between households with a diagnosis of cancer compared to those without a diagnosis of cancer ($7257 vs $9150, *p* = 0.4250). In sum, these unadjusted results suggest that OOPC might impose a financial burden on households in which members experienced a diagnosis of cancer compared to those that did not experience a diagnosis of cancer.

In Table 3, results from multivariate models that adjusted for an extensive set of variables showed that households (either the head of household or spouse if married) that reported a diagnosis of cancer had odds of 2.13 of having any OOPC than those without a diagnosis of cancer (*p* < 0.01). For households with positive amounts of OOPC, a change in the cancer diagnosis by a household member from not having a diagnosis of cancer to having a diagnosis of cancer was associated with a change of 73% in the amount of OOPC, which represented an increase of $738 over the control mean (*p* < 0.01). For any medical debt, although the unadjusted differences in the likelihood of having any medical debt between households with and without a diagnosis of cancer were not statistically different, the multivariate model found a statistically significant difference in the odds of having any medical debt when controlling for confounders between households with and without a diagnosis of cancer. Households that reported a diagnosis of cancer had odds of 1.55 of having any medical debt relative to those without a diagnosis of cancer (*p* < 0.1).

## 4. Discussion

This study shows that a diagnosis of cancer imposes a financial burden on both OOPC and medical debt on households with a diagnosis of cancer compared to those without a diagnosis of cancer.

The current study’s findings are similar to those of recent studies that showed that a diagnosis of cancer imposes a substantial financial burden on elderly Medicare beneficiaries [20], non-elderly adult cancer survivors [1,13,19] and cancer patients diagnosed as adolescents and young adults [41]. However, while the current study found that of those households who had a head or spouse (if married) with a diagnosis of cancer, 14% had medical debt and of those without a diagnosis of cancer, 12% had medical debt, other studies have found that about 28% of individuals 18 to 64 years old experience financial difficulties from a diagnosis of cancer [21]. These differences may be due to other studies using different measures of financial hardship or being conducted at the individual level while the current study was conducted at the household level. For example, Yabroff et al. [21] used a broader measure to assess the financial hardship of cancer by including individuals who ever “borrowed money or went into debt”, “filed for bankruptcy”, were “unable to cover their share of medical care costs”, or “made other financial sacrifices because of cancer, its treatment, and lasting effects of treatment” [21].

In relation to public policy, current reforms under the Affordable Care Act (ACA) of 2010 (such as Medicaid expansion in some states and subsidies for those with low incomes to buy health insurance in the marketplace) may improve the financial situation of households with a member diagnosed with cancer. Our study, once more, confirms the importance of health insurance in alleviating some of the costs associated with medical care, particularly for expensive and chronic conditions such as cancer. For instance, results showed that households that lacked health insurance had significantly higher odds of having any medical debt and higher amounts of medical debt compared to those with private insurance. On the other hand, Medicaid beneficiary households were less likely to have any OOPC and had lower amounts of OOPC than households with private health insurance. In light of these results, provisions from the ACA that increase health insurance coverage may provide relief to households in which a family member is diagnosed with cancer. Of note, the current study is not drawing conclusions on the ACA but making implications, because the analyzed data was collected during 2013 and some of the major provisions particularly the Medicaid expansion provision, the mandate and the provision of taxes and subsidies by the federal government to purchase health insurance, were not implemented until 2014. However, given the results of our study, we expect that the provisions mentioned above will be associated with a reduction in OOPC and medical debt for households in which patients are diagnosed with cancer.

Davidoff et al. [20] found an inflated incremental OOPC spending of $976 (2007 constant dollars) by elderly Medicare beneficiaries with cancer compared to their counterparts without cancer. Based on the Medical Component of the Consumer Price Index (CPI), this $976 annual incremental OOPC is equivalent to about $1152 in 2013 [42]. Our study found that having a household cancer diagnosis is associated with an incremental OOPC amount of $738 compared to households without a diagnosis of cancer. The annual incremental spending found by Davidoff et al. [20] is larger than in our study. This may be due to the subjects in the Davidoff et al. [20] study being older and experiencing greater comorbid conditions than our study subjects.

This study has important strengths and advances the literature by examining the association between a diagnosis of cancer and financial outcomes with a family unit that is composed of non-elderly members of a health insurance eligibility unit. The sample size for any OOPC, any medical debt, and amount of OOPC is large enough to achieve at least 80% power to detect a difference in means of 0.20 standard deviations with a 5% two-sided significance level. However, for the amount of medical debt for households with positive debt, the sample size of 639 households does not achieve 80% power given the difference in the means and the standard deviations between households with a diagnosis of cancer compared to those without a diagnosis of cancer. This study has some further limitations. We were unable to account for illness duration, different stages of the cancer diagnosis, and treatment cycle after the diagnosis. A lifetime prevalence of a diagnosis of cancer was used to examine the relationship between a diagnosis of cancer and financial burden. Of note, the use of lifetime prevalence of cancer as opposed to incidence might have different implications on financial burden [40]. For instance, someone with a stage IV cancer and at the end of a treatment continuum would experience a greater financial burden compared to someone who has just been diagnosed with cancer. Further, it is possible that not all reported medical bills for the survey question on medical debt would be considered as medical debt, as some patients may be undergoing treatment and may pay their medical bills later prior to becoming medical debt. Additional limitations include the diagnosis of cancer being self-reported, a sample size that prevented an analysis by cancer type (as different types of cancer and treatments may impact financial outcomes differently), and lack of data on the non-medical financial expenses that may be incurred as a result of receiving medical care (e.g., cost of child care during doctor’s appointments and cost of transportation to and from doctor’s appointments). Lastly, the study is about a correlation between a diagnosis of cancer and financial outcomes and makes no claims of any causal inference. Further studies may utilize the panel structure of the survey and compare households pre- and post-diagnosis of cancer as well as control for household wealth. Additional studies may also focus on debt in general to account for payment of medical debt by other means which may result in other types of debt.

## 5. Conclusions

This study showed that a diagnosis of cancer imposes a significant financial burden, both OOPC and medical debt, on households with a diagnosis of cancer compared to those without a diagnosis of cancer. Findings apply to all types of cancer (e.g., bladder cancer, breast cancer, colon cancer, lung cancer, prostate cancer). Future studies should investigate the effect of Medicaid coverage expansion on the financial burden of those with a diagnosis of cancer. This study and future studies would inform providers of the importance of taking cost information and cost sharing for medical treatment into account in clinical decision-making.

## Figures and Tables

**Table 1:** Weighted Summary Statistics of Dependent and Independent Variables by Diagnosis of Cancer, 2013 Panel Study of Income Dynamics [31].

Variables	Total Sample (*n* = 4407)	Any Diagnosis of Cancer (*n* = 242)	No Diagnosis of Cancer (*n* = 4165)	*p*-Value
	Means/Prop.	Means/Prop.	Means/Prop.	
**Dependent Variables**				
OOPC				
Any OOPC	0.78	0.91	0.78	0.0000
	[0.76,0.81]	[0.88,0.95]	[0.75,0.80]	
Medical Debt				
Any Medical Debt	0.12	0.14	0.12	0.4289
	[0.10,0.14]	[0.08,0.21]	[0.10,0.14]	
**Independent Variables**				
Any Diagnosis of Cancer (both the head of household and spouse if married)	0.056	1	0	
	[0.05,0.06]	[1.00,1.00]	[0.00,0.00]	
*Head of Household*				
Sex				
Female	0.24	0.18	0.24	0.0613
	[0.21,0.26]	[0.12,0.24]	[0.22,0.27]	
Male (reference)	0.76	0.82	0.76	
	[0.74,0.79]	[0.76,0.88]	[0.73,0.78]	
Age (categories)				
Age 18–34 Years (reference)	0.26	0.078	0.28	0.0000
	[0.25,0.28]	[0.04,0.12]	[0.26,0.29]	
Age 35–44 Years	0.25	0.15	0.25	0.0038
	[0.23,0.27]	[0.08,0.22]	[0.23,0.27]	
Age 45–64 Years	0.49	0.77	0.47	0.0000
	[0.47,0.51]	[0.69,0.85]	[0.45,0.49]	
Race				
White (reference)	0.74	0.89	0.74	0.0000
	[0.69,0.79]	[0.84,0.94]	[0.68,0.79]	
Black	0.14	0.049	0.14	0.0000
	[0.10,0.18]	[0.02,0.08]	[0.10,0.18]	
Hispanic	0.095	0.040	0.098	0.0044
	[0.07,0.12]	[0.01,0.07]	[0.07,0.13]	
Other	0.024	0.020	0.024	0.7409
	[0.02,0.03]	[−0.01,0.04]	[0.02,0.03]	
Marital Status				
Married (reference)	0.56	0.70	0.55	0.0015
	[0.54,0.58]	[0.61,0.78]	[0.53,0.58]	
Never Married	0.24	0.13	0.24	0.0011
	[0.22,0.26]	[0.07,0.19]	[0.22,0.27]	
Not Married ^++^	0.20	0.17	0.21	0.3330
	[0.18,0.22]	[0.11,0.24]	[0.19,0.22]	
Education				
Years of Education	14.0	14.3	14.0	0.1212
	[13.87,14.23]	[13.94,14.74]	[13.85,14.21]	
Years of Education ^2^	203.3	210.0	202.8	0.2043
	[198.62,207.89]	[198.36,221.69]	[198.20,207.49]	
*Household*				
Health Insurance Status				
Employer/Private Coverage (reference)	0.78	0.82	0.78	0.2323
	[0.76,0.80]	[0.76,0.88]	[0.76,0.80]	
Medicaid	0.047	0.040	0.047	0.6689
	[0.04,0.06]	[0.01,0.07]	[0.04,0.06]	
Uninsured	0.17	0.14	0.17	0.3584
	[0.15,0.18]	[0.08,0.20]	[0.15,0.19]	
Other CHCs				
Number of Other CHCs	0.75	1.18	0.73	0.0001
	[0.70,0.81]	[0.97,1.40]	[0.67,0.78]	
Children				
Number of Dependent Children	0.81	0.49	0.83	0.0002
	[0.75,0.86]	[0.33,0.65]	[0.77,0.89]	
Geographic Region				
Northeast (reference)	0.19	0.24	0.19	0.1654
	[0.12,0.26]	[0.13,0.34]	[0.12,0.26]	
North Central	0.27	0.24	0.27	0.3325
	[0.19,0.35]	[0.13,0.34]	[0.19,0.35]	
South	0.32	0.36	0.32	0.2175
	[0.26,0.38]	[0.26,0.47]	[0.26,0.38]	
West	0.22	0.16	0.22	0.0387
	[0.15,0.29]	[0.09,0.23]	[0.15,0.29]	
Geographic Variation				
Urban (reference)	0.67	0.60	0.67	0.1080
	[0.61,0.72]	[0.50,0.71]	[0.61,0.73]	
Rural	0.33	0.40	0.33	
	[0.28,0.39]	[0.29,0.50]	[0.27,0.39]	

Note: 95% confidence intervals are presented in brackets. Legend: Prop. = Proportions; OOPC = Out of Pocket Costs; Not Married ^++^ = Separated, Divorced or Widowed; Years of Education ^2^ = Years of Education Completed Square; CHC = Chronic Health Condition.

**Table 2:** Weighted Summary Statistics of Dependent and Independent Variables by Diagnosis of Cancer for Households with Positive Amounts of OOPC and Medical Debt, 2013 Panel Study of Income Dynamics [31].

Variables	Total Sample for Amount of OOPC > 0 (*n* = 3183)	Any Diagnosis of Cancer for Amount of OOPC > 0 (*n* = 164)	No Diagnosis of Cancer for Amount of OOPC > 0 (*n* = 3019)	*p*-Value	Total Sample for Amount of Medical Debt > 0 (*n* = 639)	Any Diagnosis of Cancer for Amount of Medical Debt > 0 (*n* = 30)	No Diagnosis of Cancer for Amount of Medical Debt > 0 (*n* = 609)	*p*-Value
	Means/Prop.	Means/Prop.	Means/Prop.		Means/Prop.	Means/Prop.	Means/Prop.	
**Dependent** **Variables**								
OOPC								
Any OOPC	1	1	1		0.79	0.84	0.78	0.4260
	[1.00,1.00]	[1.00,1.00]	[1.00,1.00]		[0.74,0.83]	[0.72,0.96]	[0.73,0.83]	
Amount of OOPC (OOPC > 0)	1060.6	1765.5	1011.1	0.0004	1196.2	1938.4	1143	0.0214
	[975.26,1146.03]	[1347.51,2183.51]	[927.40,1094.84]		[987.82,1404.61]	[1270.23,2606.62]	[920.22,1365.70]	
Log Amount of OOPC	6.08	6.59	6.05	0.0000	5.07	6.1	5	0.0343
	[6.01,6.15]	[6.36,6.83]	[5.97,6.12]		[4.72,5.42]	[5.19,7.00]	[4.62,5.37]	
Medical Debt								
Any Medical Debt	0.12	0.13	0.12	0.6902	1	1	1	
	[0.10,0.14]	[0.07,0.19]	[0.10,0.14]		[1.00,1.00]	[1.00,1.00]	[1.00,1.00]	
Amount of Medical Debt (Medical Debt > 0)	1051.2	918.6	1060.6	0.6803	9023	7257.2	9149.7	0.4250
	[714.81,1387.67]	[317.76,1519.40]	[704.31,1416.82]		[7004.93,11041.05]	[2693.55,11820.83]	[7032.23,11267.17]	
Log Amount of Medical Debt	0.94	1.04	0.94	0.6375	7.92	7.84	7.93	0.7830
	[0.79,1.10]	[0.57,1.51]	[0.78,1.09]		[7.76,8.09]	[7.19,8.49]	[7.77,8.09]	
**Independent** **Variables**								
Any Diagnosis of Cancer (both the head of household and spouse if married)	0.066	1	0		0.067	1	0	
	[0.06,0.08]	[1.00,1.00]	[0.00,0.00]		[0.04,0.09]	[1.00,1.00]	[0.00,0.00]	
*Head of Household*								
Sex								
Female	0.22	0.17	0.22	0.1182	0.35	0.3	0.36	0.6367
	[0.20,0.24]	[0.10,0.23]	[0.20,0.25]		[0.30,0.41]	[0.07,0.53]	[0.30,0.41]	
Male (reference)	0.78	0.83	0.78		0.65	0.7	0.64	
	[0.76,0.80]	[0.77,0.90]	[0.75,0.80]		[0.59,0.70]	[0.47,0.93]	[0.59,0.70]	
Age (categories)								
Age 18–34 Years (reference)	0.22	0.067	0.23	0.0000	0.34	0.075	0.36	0.0000
	[0.20,0.24]	[0.03,0.11]	[0.21,0.25]		[0.28,0.41]	[−0.01,0.16]	[0.30,0.43]	
Age 35–44 Years	0.25	0.15	0.26	0.0050	0.28	0.35	0.27	0.4583
	[0.23,0.28]	[0.07,0.23]	[0.24,0.29]		[0.23,0.32]	[0.14,0.57]	[0.22,0.32]	
Age 45–64 Years	0.53	0.78	0.51	0.0000	0.38	0.57	0.36	0.0779
	[0.50,0.55]	[0.70,0.86]	[0.48,0.53]		[0.32,0.44]	[0.32,0.82]	[0.31,0.42]	
Race								
White (reference)	0.78	0.9	0.77	0.0000	0.68	0.87	0.66	0.0090
	[0.74,0.83]	[0.85,0.95]	[0.73,0.82]		[0.61,0.75]	[0.74,1.00]	[0.59,0.74]	
Black	0.11	0.033	0.11	0.0000	0.2	0.081	0.21	0.0178
	[0.07,0.14]	[0.01,0.06]	[0.08,0.15]		[0.14,0.26]	[−0.02,0.18]	[0.14,0.28]	
Hispanic	0.087	0.043	0.09	0.0217	0.1	0.048	0.11	0.2833
	[0.06,0.11]	[0.01,0.08]	[0.06,0.12]		[0.06,0.15]	[−0.05,0.15]	[0.06,0.16]	
Other	0.023	0.022	0.023	0.9146	0.017	0	0.018	0.0070
	[0.02,0.03]	[−0.01,0.05]	[0.01,0.03]		[0.00,0.03]	[0.00,0.00]	[0.01,0.03]	
Marital Status								
Married (reference)	0.62	0.72	0.61	0.0340	0.45	0.62	0.44	0.1524
	[0.59,0.65]	[0.63,0.80]	[0.59,0.64]		[0.39,0.51]	[0.36,0.88]	[0.37,0.50]	
Never Married	0.19	0.13	0.19	0.0783	0.3	0.12	0.31	0.0734
	[0.16,0.21]	[0.06,0.20]	[0.17,0.22]		[0.23,0.36]	[−0.10,0.35]	[0.25,0.37]	
Not Married ^++^	0.19	0.15	0.19	0.2548	0.25	0.26	0.25	0.9883
	[0.17,0.21]	[0.09,0.22]	[0.17,0.22]		[0.21,0.30]	[0.02,0.49]	[0.21,0.30]	
Education								
Years of Education	14.2	14.4	14.2	0.2054	12.9	13.1	12.9	0.5267
	[14.01,14.36]	[14.00,14.84]	[13.99,14.34]		[12.61,13.13]	[12.31,13.94]	[12.57,13.13]	
Years of Education ^2^	206.8	212.2	206.4	0.3130	171.4	174.7	171.1	0.7604
	[202.15,211.44]	[199.99,224.32]	[201.83,211.00]		[165.52,177.20]	[152.14,197.26]	[164.83,177.40]	
*Household*								
Health Insurance Status								
Employer/Private Coverage (reference)	0.85	0.86	0.85	0.7435	0.59	0.67	0.59	0.3803
	[0.83,0.87]	[0.80,0.92]	[0.83,0.87]		[0.53,0.66]	[0.46,0.89]	[0.53,0.65]	
Medicaid	0.022	0.0061	0.023	0.0054	0.081	0.095	0.08	0.7672
	[0.01,0.03]	[−0.00,0.02]	[0.02,0.03]		[0.05,0.11]	[−0.00,0.19]	[0.05,0.11]	
Uninsured	0.12	0.13	0.12	0.7252	0.32	0.23	0.33	0.3269
	[0.11,0.14]	[0.07,0.20]	[0.11,0.14]		[0.26,0.38]	[0.02,0.44]	[0.27,0.38]	
Other CHCs								
Number of Other CHCs	0.81	1.16	0.79	0.0014	1.06	1.75	1.01	0.0241
	[0.75,0.87]	[0.94,1.38]	[0.72,0.85]		[0.91,1.20]	[1.09,2.41]	[0.86,1.15]	
Children								
Number of Dependent Children	0.8	0.45	0.83	0.0001	0.84	0.5	0.87	0.0137
	[0.74,0.86]	[0.27,0.62]	[0.77,0.89]		[0.73,0.96]	[0.23,0.76]	[0.74,0.99]	
Geographic Region								
Northeast (reference)	0.2	0.23	0.2	0.3307	0.11	0.12	0.11	0.9310
	[0.12,0.28]	[0.13,0.34]	[0.12,0.27]		[0.04,0.17]	[−0.11,0.34]	[0.05,0.17]	
North Central	0.27	0.24	0.27	0.3749	0.31	0.4	0.31	0.3652
	[0.19,0.35]	[0.13,0.35]	[0.19,0.35]		[0.21,0.42]	[0.17,0.62]	[0.20,0.41]	
South	0.32	0.37	0.32	0.1528	0.39	0.31	0.4	0.3926
	[0.26,0.38]	[0.26,0.47]	[0.25,0.38]		[0.30,0.49]	[0.05,0.57]	[0.31,0.50]	
West	0.21	0.16	0.21	0.0846	0.18	0.18	0.18	0.9442
	[0.14,0.28]	[0.09,0.24]	[0.14,0.29]		[0.09,0.28]	[0.00,0.36]	[0.09,0.28]	
Geographic Variation								
Urban (reference)	0.67	0.61	0.67	0.1241	0.56	0.27	0.58	0.0057
	[0.61,0.73]	[0.50,0.71]	[0.61,0.73]		[0.47,0.64]	[0.04,0.51]	[0.49,0.66]	
Rural	0.33	0.39	0.33	0.1241	0.44	0.73	0.42	
	[0.27,0.39]	[0.29,0.50]	[0.27,0.39]		[0.36,0.53]	[0.49,0.96]	[0.34,0.51]	

Note: 95% confidence intervals are presented in brackets. Legend: OOPC = Out of Pocket Costs; Not Married ^++^ = Separated, Divorced or Widowed; Years of Education ^2^ = Years of Education Completed Square; CHC = Chronic Health Condition.

**Table 3:** Results from Multivariate Models for Any Out of Pocket Costs, Amount of Out of Pocket Costs (>$0), Any Medical Debt and Amount of Medical Debt (>$0), 2013 PSID [31].

Variable	Odds Ratios		Log Amount	
	Any OOPC (*n* = 4407)	Any Medical Debt (*n* = 4407)	OOPC (*n* = 3183)	Medical Debt (*n* = 639)
Any Diagnosis of Cancer (both the head of household and spouse if married)	2.13 ***	1.55 *	1.73 ***	0.87
	[1.27,3.57]	[0.93,2.58]	[1.33,2.25]	[0.47,1.62]
Sex (Male)				
Female	1.53 ***	2.02 ***	1.19	1.28
	[1.13,2.06]	[1.32,3.08]	[0.88,1.61]	[0.83,1.98]
Age (categories) (Ref: Age 18–34 years)				
Age 35–44 Years	1.58 ***	0.77	1.11	1.35
	[1.21,2.07]	[0.53,1.12]	[0.91,1.35]	[0.91,2.00]
Age 45–64 Years	1.72 ***	0.40 ***	1.18	1.41 *
	[1.33,2.23]	[0.27,0.59]	[0.96,1.46]	[0.93,2.13]
Race (Ref: White)				
Black	0.46 ***	1.24	0.81	1.4
	[0.34,0.63]	[0.86,1.78]	[0.61,1.08]	[0.93,2.10]
Hispanic	0.87	0.79	0.66 ***	0.75
	[0.59,1.29]	[0.47,1.32]	[0.53,0.82]	[0.44,1.28]
Other	0.7	1.16	1.04	2.78 *
	[0.34,1.44]	[0.54,2.48]	[0.74,1.45]	[0.83,9.34]
Marital Status (Married)				
Never Married	0.40 ***	1.07	0.57 ***	0.77
	[0.28,0.57]	[0.70,1.64]	[0.45,0.73]	[0.46,1.31]
Not Married ^++^	0.45 ***	1.17	0.71 ***	1.12
	[0.31,0.66]	[0.69,1.98]	[0.57,0.88]	[0.70,1.79]
Education				
Years of Education	1.01	1.16	0.91	0.99
	[0.79,1.29]	[0.86,1.59]	[0.80,1.04]	[0.82,1.20]
Years of Education ^2^	1	0.99 **	1	1
	[0.99,1.01]	[0.97,1.00]	[1.00,1.01]	[0.99,1.00]
Health Insurance Status (Employer/Private Coverage)				
Medicaid	0.12 ***	1.14	0.57 **	1.77 *
	[0.08,0.18]	[0.70,1.87]	[0.38,0.88]	[0.95,3.27]
Uninsured	0.34 ***	1.77 ***	1.2	2.20 ***
	[0.25,0.47]	[1.29,2.42]	[0.94,1.54]	[1.49,3.25]
Other CHCs				
Log Number of Other CHCs	1.59 ***	2.27 ***	1.22 ***	1.57 ***
	[1.22,2.08]	[1.80,2.87]	[1.06,1.41]	[1.14,2.17]
Children				
Log Number of Children	1	1	1.41 ***	1.28
	[0.79,1.27]	[0.77,1.30]	[1.21,1.65]	[0.94,1.74]
Geographic Region (Northeast)				
North Central	0.89	1.66 **	1.31 **	2.06 **
	[0.62,1.29]	[1.11,2.47]	[1.00,1.73]	[1.08,3.93]
South	0.91	1.89 ***	1.2	1.54
	[0.63,1.31]	[1.31,2.73]	[0.95,1.52]	[0.84,2.84]
West	0.81	1.41	1.1	1.13
	[0.55,1.21]	[0.83,2.40]	[0.85,1.43]	[0.60,2.11]
Geographic Variation (Urban)				
Rural	0.96	1.41 ***	1.08	0.94
	[0.74,1.26]	[1.13,1.77]	[0.91,1.28]	[0.64,1.38]

* *p* < 0.10, ** *p* < 0.05, *** *p* < 0.01. Note: 95% confidence intervals are presented in brackets and reference groups are in parentheses. Legend: OOPC = Out of Pocket Costs; Not Married ^++^ = Separated, Divorced or Widowed; Years of Education ^2^ = Years of Education Completed Square; CHC = Chronic Health Condition.

## Data Availability

All 2013 PSID files are available from the PSID Public Data Extract Repository (http://doi.org/10.3886/E100785V1, accessed on 17 July 2017).
